# Report of a Novel *SHOX* Missense Variant in a Boy With Short Stature and His Mother With Leri–Weill Dyschondrosteosis

**DOI:** 10.3389/fendo.2018.00163

**Published:** 2018-04-10

**Authors:** Laura Lucchetti, Paolo Prontera, Amedea Mencarelli, Ester Sallicandro, Annalisa Mencarelli, Marta Cofini, Alberto Leonardi, Gabriela Stangoni, Laura Penta, Susanna Esposito

**Affiliations:** ^1^Pediatric Clinic, Department of Surgical and Biomedical Sciences, Università degli Studi di Perugia, Perugia, Italy; ^2^Medical Genetics Unit, Santa Maria della Misericordia Hospital, Perugia, Italy

**Keywords:** short stature, Leri–Weill dyschondrosteosis, novel missense mutation, *SHOX*, pediatric endocrinology

## Abstract

Heterozygous mutations in the *SHOX* gene or in the upstream and downstream enhancer elements are associated with 2–22% of cases of idiopathic short stature (OMIM #300582) and with 60% of cases of Leri–Weill dyschondrosteosis (OMIM #127300) with which female subjects are generally more severely affected. Approximately 80–90% of *SHOX* pathogenic variants are deletions or duplications, and the remaining 10–20% are point mutations that primarily give rise to missense variants. The clinical interpretation of novel variants, particularly missense variants, can be challenging and can remain of uncertain significance. Here, we describe a novel missense variant (c.1044 G>T, p.Arg118Met) in a Moroccan boy with a disproportionately short stature and without any radiological traits or bone deformities and in his mother, who had a disproportionately short stature and a Madelung deformity. This variant has not been reported to date in the updated *SHOX* allelic variant or Human Gene Mutation Databases nor is it listed as a polymorphism in the ExAC browser, dbSNP, or 1000G. This mutation was predicted to be deleterious by three different bioinformatics tools since it modifies an amino acid in a highly conserved DNA-binding domain of the SHOX protein. Based on this evidence, the patient was treated with recombinant human growth hormone.

## Background

Human growth is a multifactorial phenomenon that is controlled by nutrition, environment, endocrine factors, and many genes. Although several genes seem to be associated with short stature, only a small proportion occurs sufficiently frequently to be recognized by clinicians and radiologists (1). These genes are responsible for bone disorders that cause incorrect balances of the proliferation and differentiation of chondrocytes and pathological extensions in length and width (1). The *SHOX* gene encodes a transcription factor with a common DNA-binding domain, a so-called homeodomain, which is implicated in skeletal development. Mutations in the *SHOX* gene are a possible cause of isolated or familial short stature (2, 3).

Homozygous or compound heterozygous mutations of the *SHOX* gene or its downstream enhancer cause 75% of Langer mesomelic dysplasia (OMIM #249700), which is the more severe clinical form that includes a disproportionately short stature. Heterozygous mutations in *SHOX* or in the upstream and downstream enhancer elements are associated with 2–22% of cases of idiopathic short stature (ISS; OMIM #300582) (2, 4–6) and with 60% of cases of Leri–Weill dyschondrosteosis (LWD; OMIM #127300), which usually affects female subjects with severe manifestations (7). The most frequent mutation is the deletion of the entire or partial *SHOX* locus (i.e., 80–90% of cases), whereas point mutations appear to be less frequent (10–20%) (5, 8). The phenotype associated with heterozygous *SHOX* mutations is a continuum from milder short stature without radiological findings to disproportionately short stature with Madelung deformity. The clinical symptoms can be markedly different even among the affected members of the same family who have the same genetic defects (1, 3, 9–12). This case describes a novel *SHOX* mutation in a Moroccan boy with disproportionate short stature and his mother with disproportionate short stature and LWD.

## Case Presentation

### Clinical Report

The Ethics Committee of Umbria Region (CEVAS) approved the study, and written informed consent was obtained from the parents of the enrolled child.

**Figure 1:**
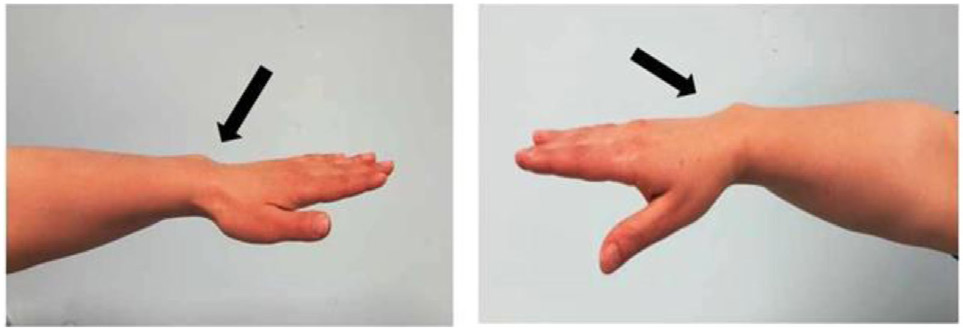
Photograph of the forearm of the proband’s mother showing a Madelung deformity and bowing of the radius (black and white arrows), which gives the hand and the wrist the appearance of a dinner fork.

The patient is a 6-year-old Moroccan boy who was born full-term after an uncomplicated pregnancy with a birth weight of 3,020 kg (−0.86 SDS) and a length of 50 cm (−0.1 SDS). He was the only child of non-consanguineous parents. His mother’s height (H) was 148.9 cm (−2.82 SDS), and his father’s H was 172.5 cm (−0.39 SDS) with a target H of 167.2 (−1.13 SDS). The mother had a disharmonic short stature (sitting H/H: 0.563 and SPAN/H: 0.953) and Madelung deformity of the forearm, which suggested a diagnosis of LWD (Figure 1).

**Figure 2:**
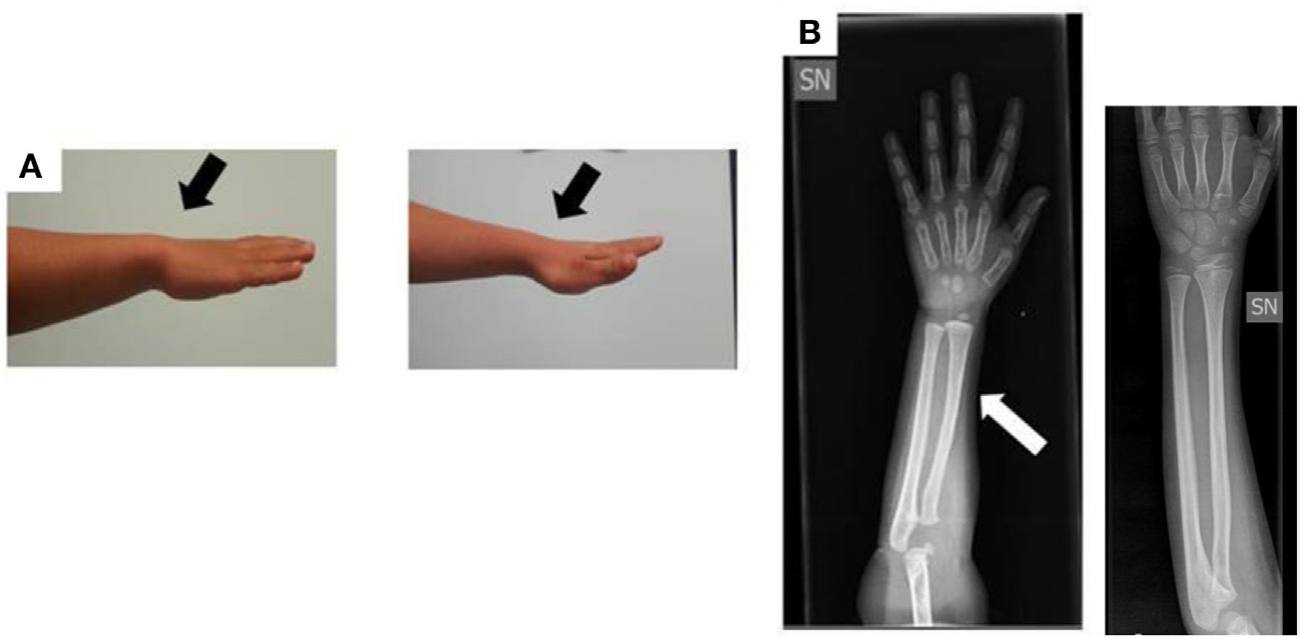
**(A)** Photograph of the forearm of the proband. No Madelung deformity is present (black arrows). **(B)** Radiograph of the forearm of the proband at the age of 3 years: the white arrow shows a mild bowing of the radius in comparison to a normal radiograph (the one without arrow).

When the patient was first referred to our Unit of Pediatric Endocrinology at the age of 2.9 years, he exhibited normal psychomotor development, his H was 86.5 cm (−1.90 SDS), his weight was 14 kg (50th percentile), and his body mass index (BMI) was 18.7 (1.38 SDS). The auxological data are expressed in SDS according to the population standard for age and gender. He was prepubertal with testes <2 mL in volume. The pubertal stage was evaluated according to the Tanner–Marshall method. The physical examination was normal with no bone deformities or dysmorphism. The bone age of hand and wrist at a chronological age of 3 years conformed to the Greulich–Pyle atlas standard for 2.4 years, and an X-ray of the wrist and forearm ruled out severe radiological deformity except for a mild bowing of the radius (Figure 2).

We re-evaluated the child after 6 and 12 months. At 3.9 years, his height was 91.3 (−2.20 SDS) with a growth velocity of 5.30 cm/year (10th p; −1.61 SDS). The Rappold’s score was 6 with a pathological sitting H/H ratio of 0.58 (normal value, <0.55) and a BMI >the 50th percentile. His arm SPAN/H ratio was normal (0.99 with a normal value >0.965), and other parameters were negative. Laboratory investigations revealed a normal hemoglobin value, and normal kidney and hepatic functions and excluded celiac disease and thyroid dysfunction. His endocrine evaluation revealed a peak growth hormone value of 4.5 ng/mL using arginine tests and 8.58 ng/mL using a clonidine test (normal value, >8) with a normal level of insulin-like growth factor-1 (IGF-1; 88 ng/mL, 50th–75th percentile), which ruled out a growth hormone deficit.

We examined the child again when he was 5.9 years old. His H was 1.99 SDS below the mean for age and sex (104.9 cm). The arm SPAN was 101.5 cm with a non-pathological arm SPAN/H ratio of 0.97. The sitting H was 59.9 cm with a distinctly supranormal sitting H/H ratio of 0.57.

### Molecular Studies

Genomic DNA was extracted from the peripheral blood of the proband and his parents following standard procedures. Thereafter, multiplex ligation-dependent probe amplification (MLPA) was performed with an SALSA MLPA P018 SHOX-G1 probe-mix kit (MRC-Holland, Amsterdam, the Netherlands) in accordance with the manufacturer’s recommendations. The results were analyzed with the Coffalyser software (MRC-Holland, Ams-terdam, The Netherlands).

A polymerase chain reaction (PCR) was performed with specific oligonucleotides to amplify the *SHOX*-coding exons (2, 3, 4, 5, and 6a) and flanking intragenic regions. The PCR products were directly sequenced using a CEQ TM8000 Genetic Analysis System (Beckman Coulter, Brea, CA, USA). The results were analyzed by comparing the sequences with those reported in Gene Bank NG_ 009385.1 and NM_004551.3 and http://grenada.lumc.nl/LOVD2/MR/home.php?select_db=SHOX (Leiden Open Variation Database, Leiden, The Netherlands).

### Results

**Figure 3:**
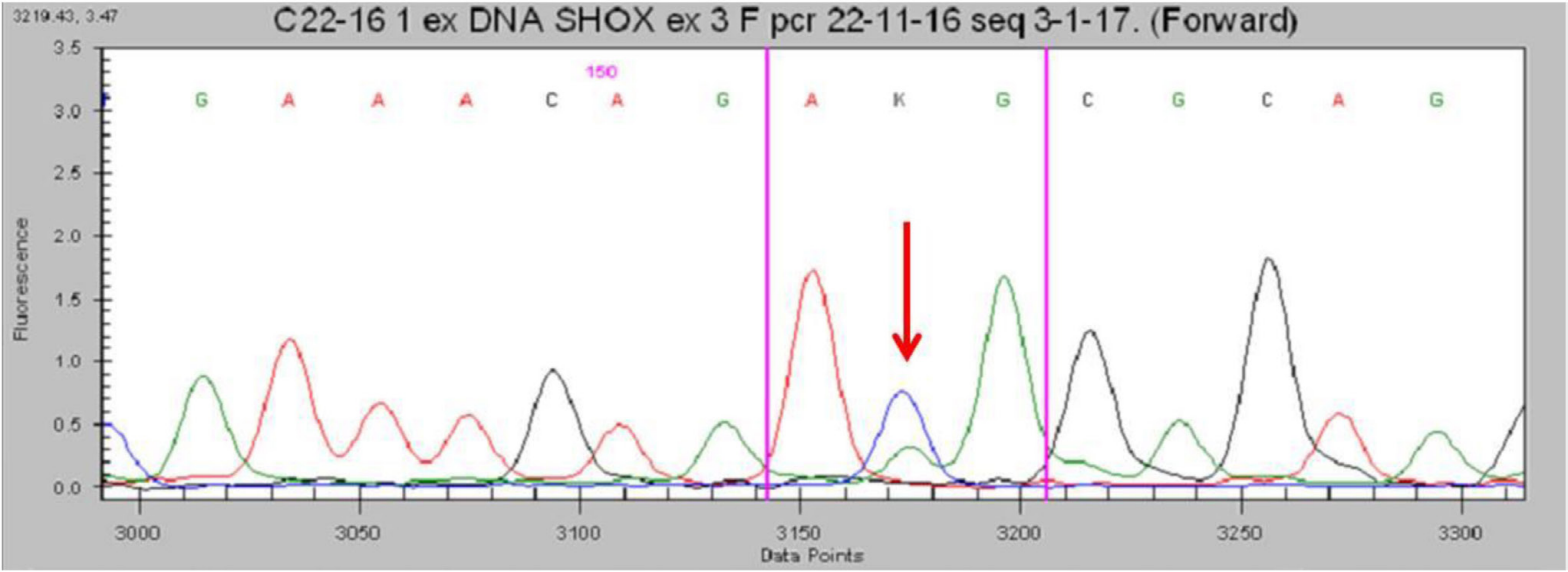
Partial electropherogram of the Sanger sequencing of *SHOX* in the patient shows (red arrow) the c.1044G>T mutation.

No deletions were found through the MLPA analysis. The *SHOX* gene sequencing revealed the heterozygous missense variant c.1044 (c.1044 G>T) in exon 3 (Figure 3). This transversion was predicted to change the inside of the homeodomain sequence from an Arginine to a Methionine in codon 118 (p.Arg118Met). This variant was found in neither the ExAC nor 1000G databases. Moreover, it has not been described in the Human Gene Mutation Database, the Human Short Stature Gene Allelic Variant Database Web Site, or the X-chromosome gene database (13, 14). The same variant was found in his mother.

After the analysis of the molecular studies, therapy with recombinant human growth hormone (rh-GH) was started according to the Italian Drug Administration (AIFA) and an auxological follow-up was initiated. The starting rh-GH dose was 0.030 mg/kg/day.

To assess the effect of rh-GH therapy, we considered the difference in height SDS, height velocity both as SDS and as cm/years. After the first 12 months of treatment, a significant increase of height was evidenced, with a height gain of +0.40 SDS (Tanner growth charts). Also the height velocity got better reaching 7.23 cm/year corresponding to +2.16 SDS for age and sex, in comparison with −2.05 SDS before treatment.

## Discussion

This study describes the clinical and molecular data of a mother–son pair who shared short stature and a novel missense *SHOX* variant. The novel point mutation described here has not been reported in the updated databases and has not been described by other authors in the literature. Consequently, the phenotypic effect of this mutation is not completely known. This variant segregates in the family reported here with short stature (proband) and LWD (mother), and the latter phenotype is very specific for *SHOX* pathogenic mutations. Moreover, this C-to-G transversion at nucleotide c.1044 (c.1044 G>T) in exon 3 is predicted to result in an amino acid alteration from Arginine to Methionine inside the homeodomain sequence in codon 118 (p.Arg118Met). This mutation affects a highly evolutionarily conserved amino acid of the homeodomain in the SHOX protein and seems to cause a loss of function by altering the secondary structure of the protein. Predictions from functional effect studies demonstrated that this mutation is probably damaging with a maximal score (PolyPhen2, SIFT, PROVEAN).

The homeodomain of *SHOX* gene mediates several key functions that include nuclear localization, DNA binding, and protein–protein interactions (15); consequently, mutations located in this region may impair these processes and lead to bone defects (1, 5, 8, 16). For these reasons, most of the point mutations (i.e., missense or nonsense mutations) that lead to ISS and LWD are located within exons 3 and 4, although they can be spread all over the gene (1, 8).

Furthermore, in the analysis of the Human Gene Mutation Database, we observed that 47 point mutations of all 88 mutations known to date are located in the areas of codons 116 and 176, which encode the homeodomain. Binder et al. described a novel missense mutation (p.Arg119Gly) located at the beginning of the homeobox (1). This mutation is a C-to-G transversion at nucleotide 355 in exon 3 that is predicted to result in a change from the charged polar amino acid Arginine to the non-polar amino acid Glycine. This mutation has been detected in two affected siblings and their mother. The siblings are a 15.9-year-old male with a disharmonic short stature (−1.77 SDS) associated with a pathological sitting H/H ratio (4.31 SDS) and a 13.3-year-old female with a severe short stature (−2.40 SDS) and pathological sitting H/H ratio (3.66 SDS). In 2002, Rappold et al. described a novel mutation located in the homeobox at codon 116. In this case, the mutation was responsible for LWD (2). In our patient, the p.ArgR118Met mutation was detected in codon 118, i.e., in the gene sequence that plays a key role in the correct function of the protein. In comparison with the previous cases, our patient has an disharmonic mild short stature (−1.90 SDS) with a pathological sitting H/H ratio (1 SDS), although his condition is probably less severe than usually reported because of his young age. Even in this case, as described in the literature for the other mutations, the main characteristics of mesomelic disproportions of the limbs and the Madelung deformity of the forearm will appear during the second decade of life (1, 3, 17). Several studies have observed that the main characteristics of mesomelic disproportions of the limbs and the Madelung deformity of the forearm develop over time, appear during the second decade of life and, occasionally, the skeletal disproportions caused by *SHOX* mutations are not yet expressed in pre-school children, especially in boys (1). In addition, in our case, the mother, who had the same mutation, had a more severe short stature and the Madelung deformity. Hence, in our case we can assess that despite the proband and his mother have the same genetic mutation, the different phenotype can be probably due both to the different age and the rule of estrogen that in female affected patients seems to worsened the clinical findings.

The challenge of finding *SHOX* mutations is due to the possibility of the treatment of all patients with *SHOX* haploinsufficiencies with rh-GH therapy. Several studies have demonstrated that rh-GH therapy improves linear growth, results in a significant increase in height SDS that reaches the lower end of the normal range by the end of the second year of therapy and results in a significant increase in IGF-1 concentration (1). According to previously auxological data reported, also in our case a significant good response to the rh-GH therapy has been evidenced.

## Conclusion

In conclusion, we found a novel point mutation that is associated with a disharmonic short stature without wrist or forearm deformities in the proband, inherited from his mother who showed LWD, a more convincing *SHOX-*related phenotype. In this case report, the genotype–phenotype correlation is not completely known, but the presence of the Madelung deformity associated with the same mutation in the proband’s mother could suggest an association between this novel mutation and *SHOX* haploinsufficiency. This case underlines the importance for clinicians in the diagnostic approach to the short stature of evaluating also children’s parents because some phenotypic characteristics of *SHOX* deficiency can develop over time.

## Consent to Publish Statement

The Ethics Committee of the University of Perugia approved the publication of this case, and both parents provided written informed consent for the publication of this manuscript.

## Ethics Statement

This study was approved by the Ethics Committee of the University of Perugia, and both parents provided written informed consent for the evaluation of themselves and the child.

## Author Contributions

LL and PP wrote the first draft of the manuscript; AmM and ES performed the laboratory analyses; AnM, MC, and AL were in charge of the patient’s follow-up; GS and LP performed the diagnosis; SE supervised the patient’s management and critically revised the paper. All the authors read and approved the final version of the manuscript.

## Conflict of Interest Statement

The authors declare that the research was conducted in the absence of any commercial or financial relationships that could be construed as a potential conflict of interest.
